# Clinical Evaluation of a Quantitative Imaging Biomarker Supporting Radiological Assessment of Hippocampal Sclerosis

**DOI:** 10.1007/s00062-023-01308-9

**Published:** 2023-06-26

**Authors:** Michael Rebsamen, Baudouin Zongxin Jin, Tomas Klail, Sophie De Beukelaer, Rike Barth, Beata Rezny-Kasprzak, Uzeyir Ahmadli, Serge Vulliemoz, Margitta Seeck, Kaspar Schindler, Roland Wiest, Piotr Radojewski, Christian Rummel

**Affiliations:** 1 Support Center for Advanced Neuroimaging (SCAN), University Institute of Diagnostic and Interventional Neuroradiology, Inselspital, Bern University Hospital, University of Bern, Freiburgstrasse 10, 3010 Bern, Switzerland; 2 Graduate School for Cellular and Biomedical Sciences, University of Bern, Bern, Switzerland; 3 Sleep-Wake-Epilepsy-Center, Department of Neurology, Inselspital, Bern University Hospital, University of Bern, Bern, Switzerland; 4 University Institute of Diagnostic and Interventional Neuroradiology, Inselspital, Bern University Hospital, University of Bern, Bern, Switzerland; 5 EEG and Epilepsy Unit, Department of Clinical Neurosciences, Geneva University Hospitals and Faculty of Medicine, University of Geneva, Geneva, Switzerland; 6 Swiss Institute for Translational and Entrepreneurial Medicine, sitem-insel, Bern, Switzerland

**Keywords:** Epilepsy, HS, MRI, Neuroradiology, Morphometry, Quantitative Reporting

## Abstract

**Objective:**

To evaluate the influence of quantitative reports (QReports) on the radiological assessment of hippocampal sclerosis (HS) from MRI of patients with epilepsy in a setting mimicking clinical reality.

**Methods:** The study included 40 patients with epilepsy, among them 20 with structural abnormalities in the mesial temporal lobe (13 with HS). Six raters blinded to the diagnosis assessed the 3T MRI in two rounds, first using MRI only and later with both MRI *and* the QReport. Results were evaluated using inter-rater agreement (Fleiss’ kappa $k$) and comparison with a consensus of two radiological experts derived from clinical and imaging data, including 7T MRI.

**Results:**

For the primary outcome, diagnosis of HS, the mean accuracy of the raters improved from 77.5% with MRI only to 86.3% with the additional QReport (effect size $d=1.43$). Inter-rater agreement increased from $k=0.56$ to $k=0.72$. Five of the six raters reached higher accuracies, and all reported higher confidence when using the QReports.

**Conclusion:**

In this pre-use clinical evaluation study, we demonstrated clinical feasibility and usefulness as well as the potential impact of a previously suggested imaging biomarker for radiological assessment of HS.

The online version of this article (10.1007/s00062-023-01308-9) contains supplementary material, which is available to authorized users.

## Introduction

Hippocampal sclerosis (HS) is the most frequently observed pathology in treatment-refractory mesial temporal lobe epilepsy [1]. For detecting such structural epileptogenic lesions, magnetic resonance imaging (MRI) with appropriate protocols is pivotal [2–5]. Radiological signs of HS include volume loss and T2/FLAIR signal alterations [6], whereas a loss of the hippocampal internal architecture is difficult to demarcate on 3T imaging [7]. The radiological interpretation from MRI is predominantly a visual task by qualitatively assessing the images, although quantitative volumetry has been generally recommended [8]. Ultra-high-field (UHF) MR imaging at 7 Tesla has shown promising results in epilepsy [9–11], e.g., better visualization of the hippocampal internal architecture [12]. Since 2017, 7T MRI has been cleared for clinical applications and implemented beyond research setting in neuro – and musculoskeletal imaging^1^. The available evidence and current consensus recommendations support the use of 7T MRI in patients with epilepsy with specific clinical questions [13, 14]. However, only a minority of patients will have access to 7T imaging due to the limited availability of the devices and high costs.

The literature contains a plethora of suggestions for automated methods to analyze MRI in epilepsy, increasingly by employing artificial intelligence [15]. Such techniques are often evaluated by assessing the accuracy against a ground truth [16–19], or contrasting accuracies with radiological reading alone [18, 20]. Given that medical decision support tools are usually intended to be used as supportive information for radiologists and not as a replacement, evaluation should be performed accordingly. However, even among commercial products with CE/FDA label, only a minority have tested and demonstrated clinical efficacy [21–23] as this is currently not a requirement for CE/FDA clearance.

The quantitative neuroradiology initiative (QNI) [24] has proposed a framework comprising six steps for the technical and clinical validation necessary to embed automated image quantification software (QReports) into the clinical neuroradiology workflow. Likewise, [21] described six levels of efficacy to assess the contribution of automated tools in a diagnostic process, ranging from technical efficacy in level I over diagnostic thinking (level III) and therapeutic (IV) efficacy to societal effect in level VI.

An imaging biomarker for HS using cross-sectional area and T2 relaxometry profiles along the hippocampal posterior-anterior axis was described by [25]. In a subsequent evaluation study, [26] reported increased accuracies with a very large effect size ($d=1.23$ for neuroradiologists) when using the additional QReports.

We have previously proposed an imaging biomarker to support the radiological assessment of HS [19]. In brief, it is based on accurate segmentation from T1-weighted MRI using deep-learning (DL) [27, 28] from which the surface-to-volume ratio of the hippocampi is calculated. The metrics are presented in a graphical report allowing direct interpretation by radiologists. A standalone evaluation against a ground truth revealed higher robustness and accuracies compared to non-DL based methods. In the present clinical validation study, we extend the evaluation by quantifying the impact when used to complement and inform expert assessment. MRI from 40 patients with epilepsy were assessed by six raters with and without additional QReports for the presence of HS and hippocampal volume abnormalities. Owing to the increased attention of amygdala enlargement in TLE [29, 30], the raters were asked to estimate amygdala volume abnormalities as well.

## Materials and Methods

### Patient Cohort

A total of 40 patients with an established diagnosis of epilepsy were included in this evaluation study, all examined with both 3T and 7T MR imaging at the Bern University Hospital (Inselspital) between August 2019 and May 2022. Patients were referred to 7T imaging during phase I or phase II evaluation [31]. Among these patients were 20 cases with radiological abnormalities in the mesial temporal lobe, thereof 13 who fulfilled the imaging criteria of HS. Additional 20 cases were added to the cohort with no known abnormalities in the mesial temporal lobe, resulting in a total of 40 cases (demographic details listed in Table S1).

A radiological *“ground truth”* for the presence of radiological signs of hippocampal sclerosis was established by consensus of two experienced imaging experts (R. W., 7 years experience in neurology/epileptology and 20 years in neuroradiology, and P. R., 10 years experience in neuroimaging, expert in 7T neuroimaging), taking into account all available clinical information including the 7T imaging and quantitative information from morphometry as recommended by [8].

MRI were acquired on 7T and 3T scanners from Siemens following the HARNESS-MRI protocol recommendations [4] which includes high-resolution 3D isotropic T1 and FLAIR, and 2D sub-millimeter T2 acquired perpendicular to the long axis of the hippocampus.

### Quantitative Reports

The QReports were generated based on the T1-weighted sequences of 3T MRI using the previously described method [19]. The publicly available version of DL+DiReCT (https://github.com/SCAN-NRAD/DL-DiReCT) was used for anatomical segmentation [27] from which volumes and surface-to-volume ratios were extracted.

A report comprises four parts as shown in Fig. S2. First, the hippocampal surface-to-volume ratios from both hemispheres are plotted against each other. In this display, symmetric hippocampi tend to appear close to the diagonal line. In addition, the volumes of the hippocampi and amygdalae are shown in a similar scheme. Lastly, a 3D rendering of the hippocampus segmentations is presented.

### Neuroradiological Rating

**Fig. 1:**
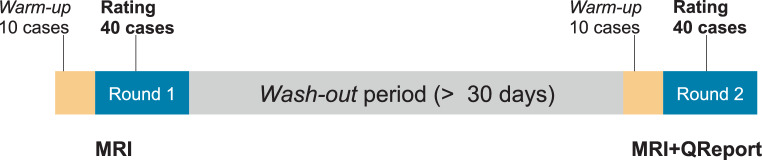
Study design: The raters assessed all 40 cases twice, first using MRI only and after a wash-out period using MRI and QReports

Six MDs with different specialization and levels of experience participated as raters in the study, among them three neurologists (two specialized in epilepsy), two neuroradiologists and one radiologist in training (Table 1). The raters assessed all 40 cases twice: in the first round using only the MRI and in the second round using both the MRI and the quantitative reports (Fig. 1). Blinded to the exact diagnosis at referral and clinical information, the raters were instructed to assess the mesial temporal structures of these patients with epilepsy. Findings were captured in a structured form (Fig. S1) and included the presence of radiological appearance of hippocampal sclerosis, as well as volume asymmetries and abnormalities of hippocampus and amygdala. For each question, the raters’ confidence scores from 1 (“not at all”) to 5 (“very confident”) were recorded.

The anonymized MR images were assessed directly in the viewer of the clinical picture archiving and communication system (PACS) (Sectra IDS7, version 23.1; Sectra AB, Linköping, Sweden), allowing the raters to work in their routine environment. Besides the MRI, only information about the sex and age of the patient was available to the raters.

The second assessment was performed after a break of at least 30 days (*“wash-out”* period) using both, the MRI *and* the QReports. Before starting their assessments, the raters received a detailed introduction and explanation of the quantitative reports. They were informed that the cases and corresponding reports were not *pre-selected*, i.e., reflect clinical reality and might, therefore, potentially also contain false positives/negatives. Raters were advised to first look at the MRI and then the QReport and that the final decision shall be made based on all available information as well as on their individual experience. Although the cases were identical to the first round, they were presented in a different random order.

Both rounds were preceded by a *warm-up* phase in which the raters assessed a different set of 10 cases according to the rating procedures described above. The purpose of this phase was to identify any ambiguities resulting from the procedure, uncover potential technical issues, and for the raters to become familiar with the procedure and flatten their learning curves before the actual rating. The raters received no feedback on their performance, neither after the *warm-up* phases nor after the first round.

### Statistical Analysis

We compared the categorical ratings to the ground truth and calculated per rater accuracies for each round (a rating was considered as correct only if identical to the ground truth, including lateralization). Differences in accuracies between the two rounds were quantified using effect sizes. Effect sizes were reported using Cohen’s $d$ [32], considering $d> 0.8$ as *large* [33] and $d> 1.2$ as *very large* effect sizes [34]. Inter-rater agreements were calculated using Fleiss’ kappa $k$7 [35, 36].

Volume asymmetries were quantified by calculating an asymmetry index:

1 $$AI(lh,rh)=\frac{lh-rh}{lh+rh}$$

where $lh$ and $rh$ represent the volumes measured on the left and right hemisphere, respectively.

Statistical analyses were performed in *R* version 4.2.1 [37] with the packages *effsize* [32] for Cohen’s $d$ and *irr* [36] for Fleiss’ kappa. Plots of the reports were created with *ggplot2* [38] and the renderings of the hippocampi with the *freeview* tool from FreeSurfer 7.0 [39].

## Results

**Fig. 2:**
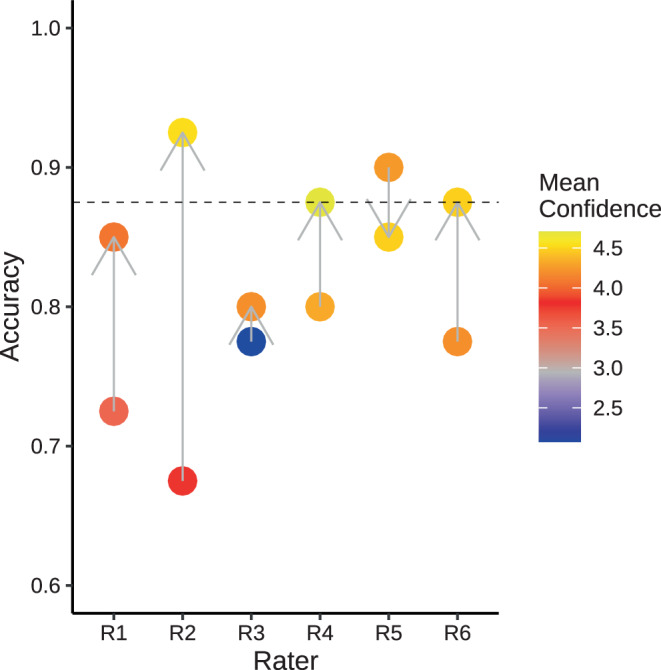
Accuracies for all six raters. The arrow points from the first round (MRI only) to the second round (MRI+QReport). The horizontal dashed line indicates accuracy of the predictions from the QReports when thresholded at three standard deviations

Including the warm-up phases, a total of 600 examinations with $\sim 1800$ MRIs were assessed, and 3600 data points were recorded by the six raters altogether (6 raters, 50 cases with 2 readings, at least 3 images per examination, 6 answers in the reporting form). The mean accuracy across all six raters regarding presence of HS was 77.5% in the first round with the availability of MRI only (Table 1). Five out of six raters reached a higher accuracy in the second round with the additional QReport available, resulting in a mean accuracy of 86.3% (Fig. 2). The accuracies improved by a very large effect size of $d=1.43$. All raters perceived a higher confidence of their rating in the second round. In contrast, if one would use the QReports alone with three standard deviations (SD) as the decision boundary, an accuracy of 87.5% would result (Fig. S3). The presence of HS was overestimated by the raters in the first round (in total 36 false positive [FP] ratings and 16 false negative [FN] ratings pooled across all raters), with a more balanced ratio in the second round (13 FP, 17 FN), as depicted in the pooled confusion matrices (Fig. S11).

**Fig. 3:**
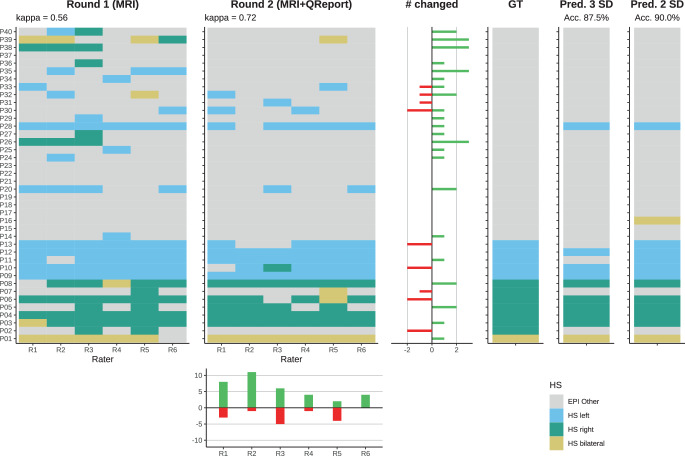
Visualization of all individual ratings (first two panels) compared to ground truth (GT) and prediction from the QReport (right panels) based on two and three standard deviations (SD). For each of the 40 cases, the number of times the raters changed their decision is depicted (# changed) with green bars where the change was in agreement with ground truth and red otherwise. Corresponding changes per rater are shown below round 2. Note: The cases were sorted by diagnosis to improve the clarity of this visualization but were presented to the raters in random order

**Fig. 4:**
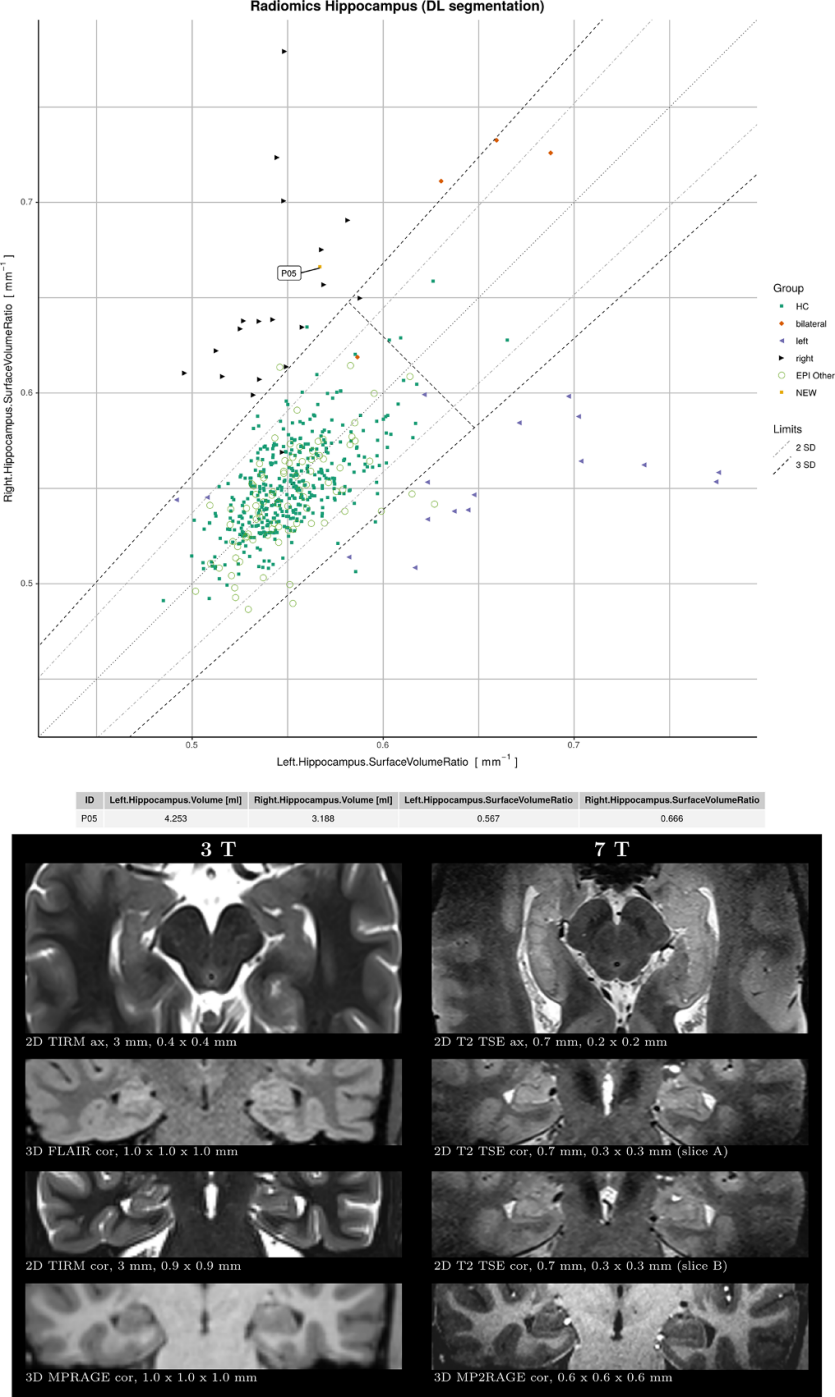
Example of patient *P05* with *HS right*. The main report is shown on top, depicting the surface-to-volume ratio of the hippocampi and below relevant images from 3T and 7T MRI. Only 3T MRI was available to the raters, whereas 7T MRI served to generate the ground truth. In the first round (MRI only), 2/6 raters classified this case as *HS right*, whereas in the second round (MRI+QReport) 4/6 raters classified it correctly. Case summary: A 26-year-old male patient with pharmacoresistant epilepsy referred for 7T MRI with strong suspicion of hippocampal sclerosis on the right side based on clinical characteristics, previous 3T MRI, FDG-PET and SPECT, including SISCOM (*Subtraction ictal SPECT coregistered to MRI*) analysis, for evaluation of the presence of other structural abnormalities in the right hemisphere. The hippocampal internal architecture (HIA) is not identifiable on 3T, whereas on 7T, the HIA is visibly intact in the left hippocampus (appearing on the right side of the image) but lost on the right side (cf. axial and coronal 2D T2 TSE 7T)

Independent of the ground truth, inter-rater agreement improved from $k=0.56$ to $k=0.72$ (Fig. 3). We have observed a tendency of the neurologists (R1–R3) to change their assessment more frequently than the neuroradiologists (R4–R6). A qualitative example of the case *P05* is shown in Fig. 4, where two raters changed their decision in the second round. Additional cases are discussed in the Appendix: Case P13, where two raters erroneously changed their rating (Fig. S4), possibly because the QReport was ambiguous with measures lying between two and three SD, P38 *without HS* that was erroneously classified as *HS right* by three raters in the first round (Fig. S5), and P28 as an example where additional clinical context is crucial for establishing a diagnosis (Fig. S6).

**Table 1:** Overview of the six raters. R1–R3 are neurologists, R4 a radiologist in training and R5–R6 neuroradiologists. Accuracy (Acc.) is calculated by comparing the exact rating (normal / HS left / HS right / HS bilateral) to the *ground truth*. Rater confidence (Conf.) is the mean over all 40 cases

Rater	Experience [years]			Round 1		Round 2		
	Neurology	Radiology	Neuroradiology	Acc. [%]	Conf.	Acc. [%]	Conf.	
R1	1.5	0	0	72.5	3.53	85.0	4.08	
R2	7.5	0	1	67.5	3.78	92.5	4.53	
R3	1.5	0	0.5	77.5	2.08	80.0	4.20	
R4	0	0	1	80.0	4.33	87.5	4.70	
R5	0	5	4	90.0	4.25	85.0	4.48	
R6	0	4	6	77.5	4.20	87.5	4.48	
Mean				77.5		86.3		$d=1.43$

Inter-rater agreement for hippocampal volume abnormalities increased from $k=0.50$ in the first round to $k=0.63$ in the second round (Figs. S7–8). Also for the amygdala, where inter-rater agreements for volume were generally much lower, they increased from $k=0.16$ to $k=0.28$ (Figs. S9–S10). On average, the raters reported an abnormality of the amygdala volumes in 30.4% of the cases in the first round, whereas this fraction reduced to 16.7% in the second round using the QReports.

## Discussion

This study investigated the use of quantitative reports (QReports) along with MRI for radiological assessment of hippocampal sclerosis (HS). The cohort comprised 40 patients with epilepsy, all examined using both 3T and 7T MRI with dedicated epilepsy protocols. Six qualified raters examined all cases twice, first using MRI only and after a *“wash-out”* period using both MRI and QReports. Mean accuracy for the primary rating of HS as well as inter-rater agreement among the raters improved by using the additional QReport. For accuracy, the improvement from 77.5% to 86.3% had very large effect size.

In agreement with the findings by [26] who performed a comparable study with a different imaging biomarker [25], we have observed a similar trend-level improvement of the diagnostic accuracies with a very large effect size. This suggests that these results are likely robust, and statistical significance is mainly a matter of the small number of raters in both studies.

Overall, we have observed a lower accuracy (77.5% in the first round) compared to the study of [26] (87.5%). This difference might be explained by the high complexity of our cases. The 7T MRI examinations of all patients were clinically indicated during detailed phase I or phase II evaluation [31] because the previous 3T MRI yielded equivocal results that did not answer all clinical questions.

Neurologists changed their decision more frequently than neuroradiologists during the second rating round when the QReports were available. This might indicate that radiologists with more experience in visual MRI interpretation are more confident in their assessment. No apparent dependence of the results on the seniority of the raters was noticed for the primary diagnosis. For the hippocampal volume asymmetries, more experienced raters tended to change their rating less frequently (cf. Fig. S7). Although of no statistical significance, it is noteworthy that a neurologist (with several years of experience in epileptology) reached the highest accuracy overall in the second round with the QReports.

We intentionally included not only experienced neuroradiologists in this study. Tools available to support MRI interpretation with quantitative evaluation are designed to be also used in primary and secondary care centers where images might be interpreted by radiologists and neurologists with regular training. On the other hand, clinical epileptologists in tertiary centers might use such tools as assistance in image interpretation.

Estimating amygdala volume abnormalities is a difficult task, as indicated by the low inter-rater agreements. The high fraction of 30.4% reported abnormalities in the first round might indicate an overestimation by the raters owing to the study setting. As we explicitly requested for an estimation, raters might have reported minor perceived asymmetries that would otherwise be described less frequently in a routine medical report. Nevertheless, by using the QReports the inter-rater agreement increased and the number of abnormalities decreased (16.4%). These findings suggest that results based on pure visual assessment of amygdala enlargement [29] should ideally be complemented by quantitative methods.

The investigated imaging biomarker is based on a previously proposed metric along with the QReport for communicating the results [19]. Artificial intelligence (AI) is used for high-quality anatomy segmentation, but not to predict a diagnosis directly. Instead, the surface-to-volume ratio is derived from the segmentation and depicted in a quantitative report. These results are *interpretable*, making it particularly suited as complementary information for expert reading and mitigating the *black-box* challenge of AI-based systems [40].

An abundance of learning-based methods for classification of hippocampal sclerosis from MRI [17, 18, 41–46] stands in stark contrast to scarce clinical evaluations [21]. While the importance of scrutinizing the standalone performance of such imaging biomarkers is undisputed, estimating the potential for a future translation into clinical applications requires an assessment of the diagnostic efficacy in a setup mimicking clinical routine. Because the implementation of a decision support system is most likely alongside a radiologist (i.e., *“human vs. human + machine”* and not *“human vs. machine”*), we have designed this evaluation study as a *level 3* [21] assessment accordingly.

### Limitations

A radiological *ground truth* reflecting the presence of radiological signs of hippocampal sclerosis was established using all available clinical information, including 7T imaging by consensus of two experts. While a histopathological diagnosis is commonly seen as the gold standard [6] for stand-alone evaluation of machine performance, the aim of this study was to assess the impact of using QReports on the radiological finding rather than the diagnostic yield. Importantly, while putative HS negative cases can rarely be examined by histology [47], the availability of 7T imaging in all 40 cases likely contributed to an improved radiological diagnosis [11], especially in these negative cases where histological confirmation is seldom available.

It is worth considering whether the raters have overestimated the occurrences of HS because they were explicitly requested to examine the MRI for the presence of HS. To a certain extent, however, this reflects the clinical reality, where referring physicians frequently ask neuroradiologists to explicitly review mesiotemporal structures in cases of semiology consistent with MTLE. Overestimation of HS in clinical routine can potentially also be due to intensity asymmetries caused by the scanner, leading to non-biological artifacts that are misinterpreted by the readers [48].

The chosen study design might still have limitations. Despite a warm-up phase preceding each round and a *wash-out* period of at least 30 days, during which the raters worked in clinical routine, we cannot exclude with certainty the possibility of some learning effects. Such a bias might be mitigated by a cross-over design where half of the cases are assessed using the QReport already in the first round (and with MRI only in the second round). Ideally, the differences between the two rounds could be contrasted to an intra-rater variability. However, this would require the raters to assess each case multiple times under identical conditions, increasing the risk of memorizing cases and significantly prolonging the study due to multiple *wash-out* periods. Most likely no study design can address all possible issues. Instead of fine-tuning the design of a pre-use evaluation study in conditions mimicking clinical reality, we suggest to increase the effort to monitor, scientifically evaluate and communicate the effect of using this or similar techniques in routine diagnostics (cf. the Outlook section).

We acknowledge the limitations of evaluating our own method. Although the assessment was performed on 40 new cases not used in our previous study [19], and none of the raters were involved in the development of the method, an unbiased evaluation should ideally be performed by an independent group [49].

### Outlook

Following the quantitative neuroradiology initiative (QNI) [24] framework, the previous publication [19] covered steps 1‑3 (i.e., identify clinical need and appropriate imaging biomarker / method for automated analysis / communication via QReports), whereas the present study corresponds to the fourth step (technical and clinical validation pre-use). The fifth step would be an integration into the clinical reporting workflow. This next step might also reveal whether the user behavior changes over time when the radiologists gain more experience interpreting the reports and learn how to integrate this additional information into their decision, such as interpreting the QReports as a continuum and not as a binary decision (cf. case P13 in Fig. S2). Incorporation of a broad spectrum of clinical, diagnostic and, increasingly, quantitative information fits well into the concept of establishing an epilepsy characterization based on converging evidence.

## Conclusions

Additional quantitative reports supporting the radiological assessment of hippocampal sclerosis decreased the inter-rater variability of raters compared to visual interpretation of 3T MR images alone. With the QReports, an increased mean accuracy by a very large effect size was observed when comparing the diagnosis to a consensus derived from 7T imaging.

### Supplementary Information

Supplementary Materials for Clinical Evaluation of a Quantitative Imaging Biomarker Supporting Radiological Assessment of Hippocampal Sclerosis

## Data Availability

DL+DiReCT, the morphometry tool for segmentation and extraction of the radiomics measures is publicly available, including trained models for non-enhanced and contrast-enhanced T1-weighted MRI (https://github.com/SCAN-NRAD/DL-DiReCT).

## Footnotes

1 FDA news release, 2017. https://www.fda.gov/news-events/press-announcements/fda-clears-first-7t-magnetic-resonance-imaging-device.
