# Supplementary material for: Clinical Evaluation of a Quantitative Imaging Biomarker Supporting Radiological Assessment of Hippocampal Sclerosis
Source: Clin Neuroradiol. 2023 Jun 26;33(4):1045–53. doi: 10.1007/s00062-023-01308-9 (PMC10654177; doi:10.1007/s00062-023-01308-9)
Supplement: Supplementary file 1 — Supplementary Materials for Clinical Evaluation of a Quantitative Imaging Biomarker Supporting Radiological Assessment of Hippocampal Sclerosis [file 62_2023_1308_MOESM1_ESM.pdf]

Supplementary Materials for
Clinical Evaluation of a Quantitative Imaging Biomarker supporting Radiological Assessment of Hippocampal Sclerosis

---

Group	# Subjects	Mean age in years (range)	%Female
Epilepsy	40	31.2 (10.4-62.3)	50.0%
HS negative	27	28.9 (10.4-62.3)	63.0%
Hippocampal Sclerosis (HS)	13	36.0 (15.5-57.5)	23.1%
left	5	39.7 (15.5-57.5)	20.0%
right	7	33.6 (18.8-55.5)	28.6%
bilateral	1	34.2 (34.2-34.2)	0.0%

Table S1: Demographic information for the patient cohort. Indented groups show a subset of parent line.

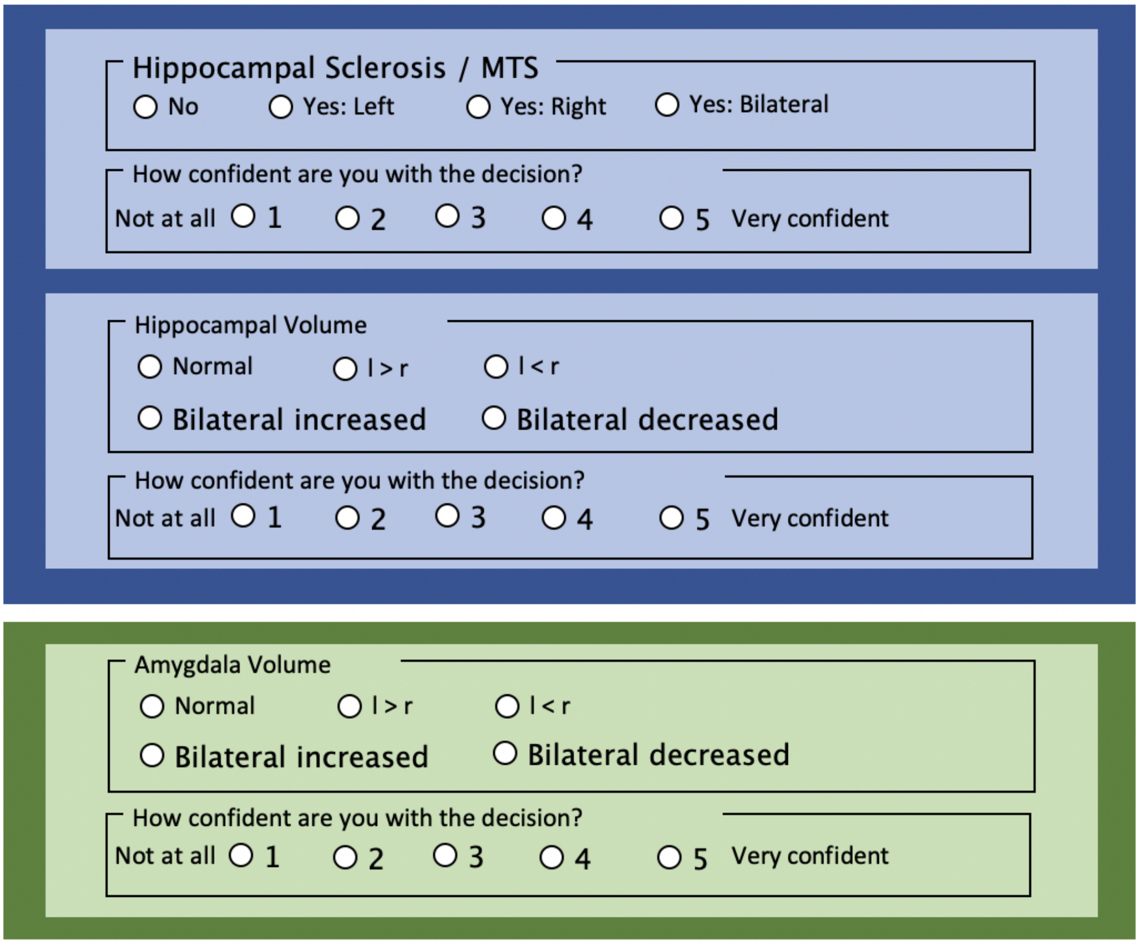
Hippocampal Sclerosis / MTS
No   Yes: Left   Yes: Right   Yes: Bilateral
How confident are you with the decision?
Not at all 1   2   3   4   5 Very confident

Hippocampal Volume
Normal   l > r   l < r
Bilateral increased   Bilateral decreased
How confident are you with the decision?
Not at all 1   2   3   4   5 Very confident

Amygdala Volume
Normal   l > r   l < r
Bilateral increased   Bilateral decreased
How confident are you with the decision?
Not at all 1   2   3   4   5 Very confident

Figure S1: Form to record the assessments of the raters.

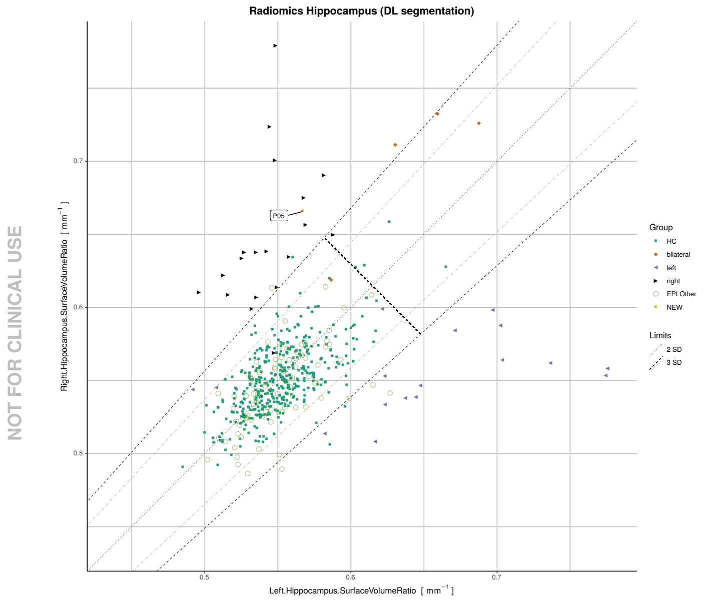

ID	Left.Hippocampus.Volume [ml]	Right.Hippocampus.Volume [ml]	Left.Hippocampus.SurfaceVolumeRatio	Right.Hippocampus.SurfaceVolumeRatio
P05	4.253	3.188	0.567	0.666

DL+DiReCT, (c) www.scancore.org

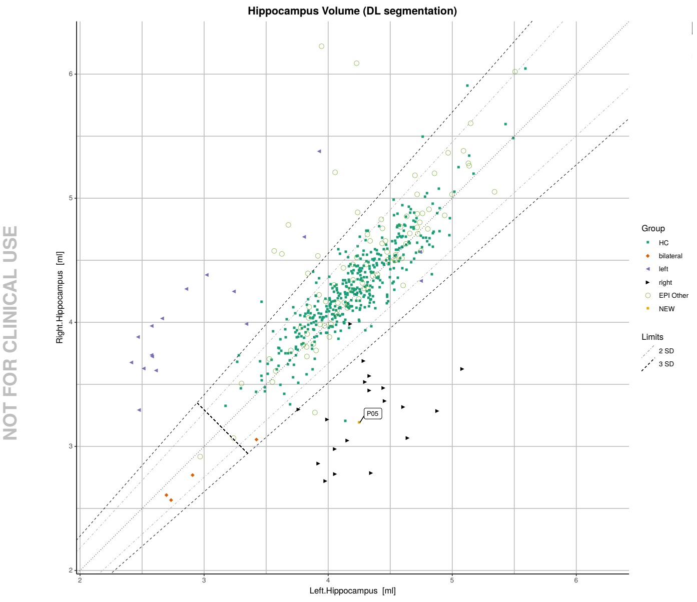

ID	Left.Hippocampus.Volume [ml]	Right.Hippocampus.Volume [ml]
P05	4.253	3.188

DL+DiReCT, (c) www.scancore.org

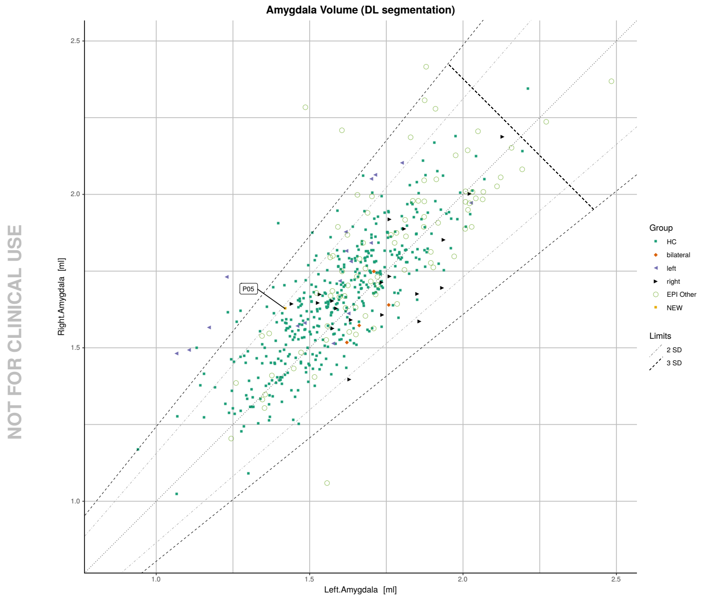

ID	Left.Amygdala.Volume [ml]	Right.Amygdala.Volume [ml]
P05	1.422	1.626

DL+DiReCT, (c) www.scancore.org

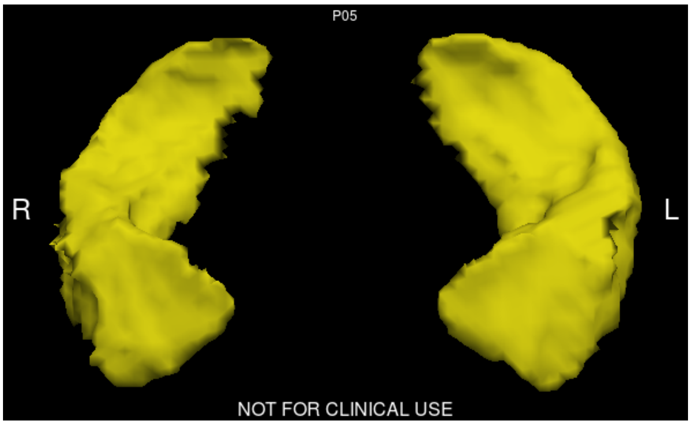

Figure S2: Sample quantitative report (QReport) from case *P05* comprising four pages. The surface-to-volume ratio of the hippocampi followed by the volumes of hippocampi and amygdalae and a 3D rendering of the hippocampi is shown. Measures from the left hemisphere on the x-axis are plotted against the right hemisphere (y-axis). The current case is highlighted respectively, while healthy controls (HC) and other epilepsy cases are shown as a reference along with exemplary cases of HS left/right/bilateral (these reference/exemplary cases are unrelated to the current study). Dashed lines represent two and three standard deviations (SD) of the healthy controls.
In the primary plot of the surface-to-volume ratio, HC and epilepsy without HS tend to cluster along the diagonal while left-sided HS tend to appear in the lower right triangle, right-sided HS on the upper left, and the bilateral cases toward the upper right corner, indicating that in contrast to the volume, the surface-to-volume ratio increases in the presence of HS.

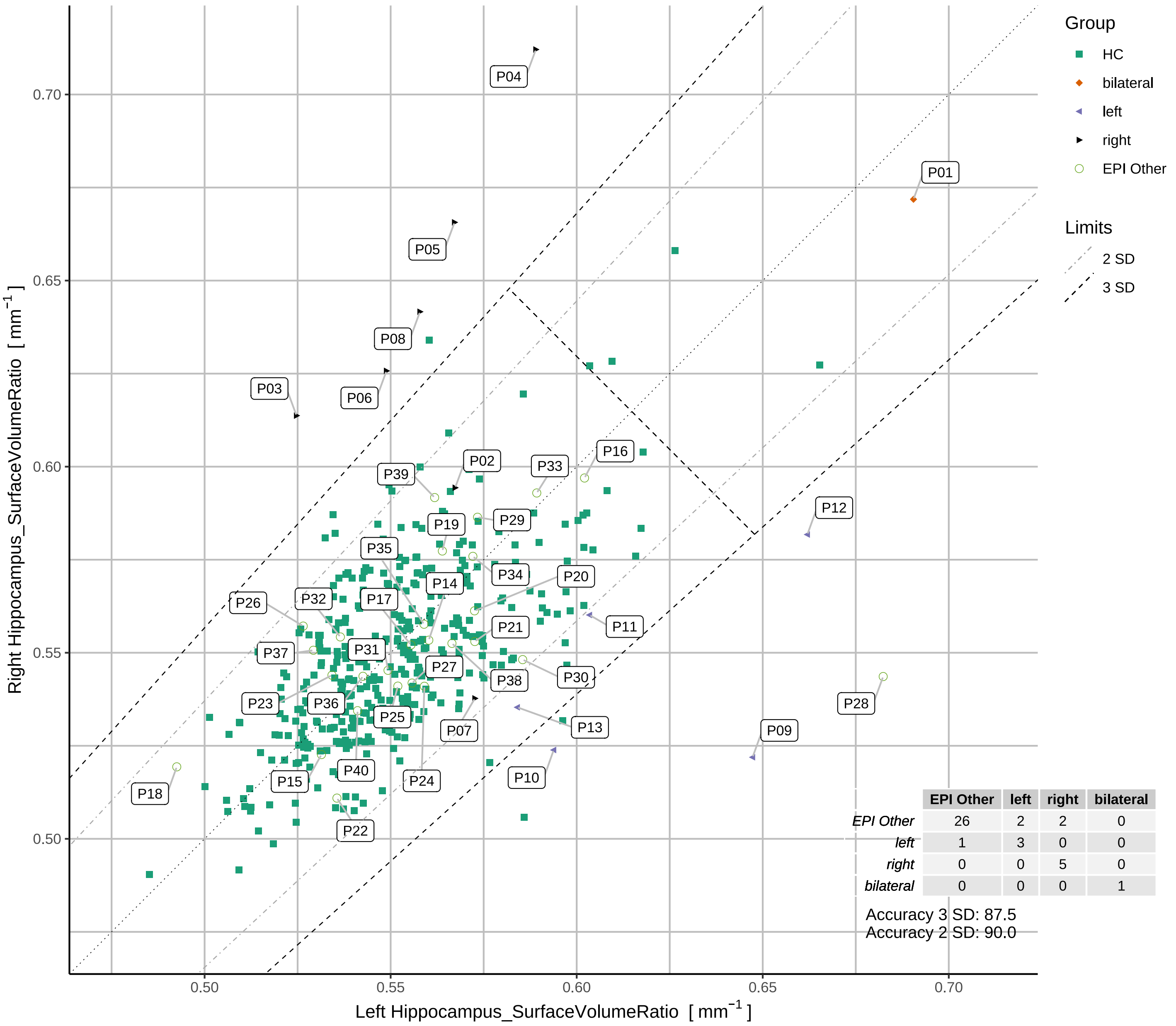

	EPI Other	left	right	bilateral
EPI Other	26	2	2	0
left	1	3	0	0
right	0	0	5	0
bilateral	0	0	0	1

Figure S3: Report of surface-to-volume ratios of all 40 cases of the study. Symbols represent ground truth derived from clinical data and 7T imaging (HS left/right/bilateral or *EPI Other* for non-HS). The confusion matrix depicts predictions based on thresholding at three standard deviations (SD) in the rows vs. ground truth in the columns.

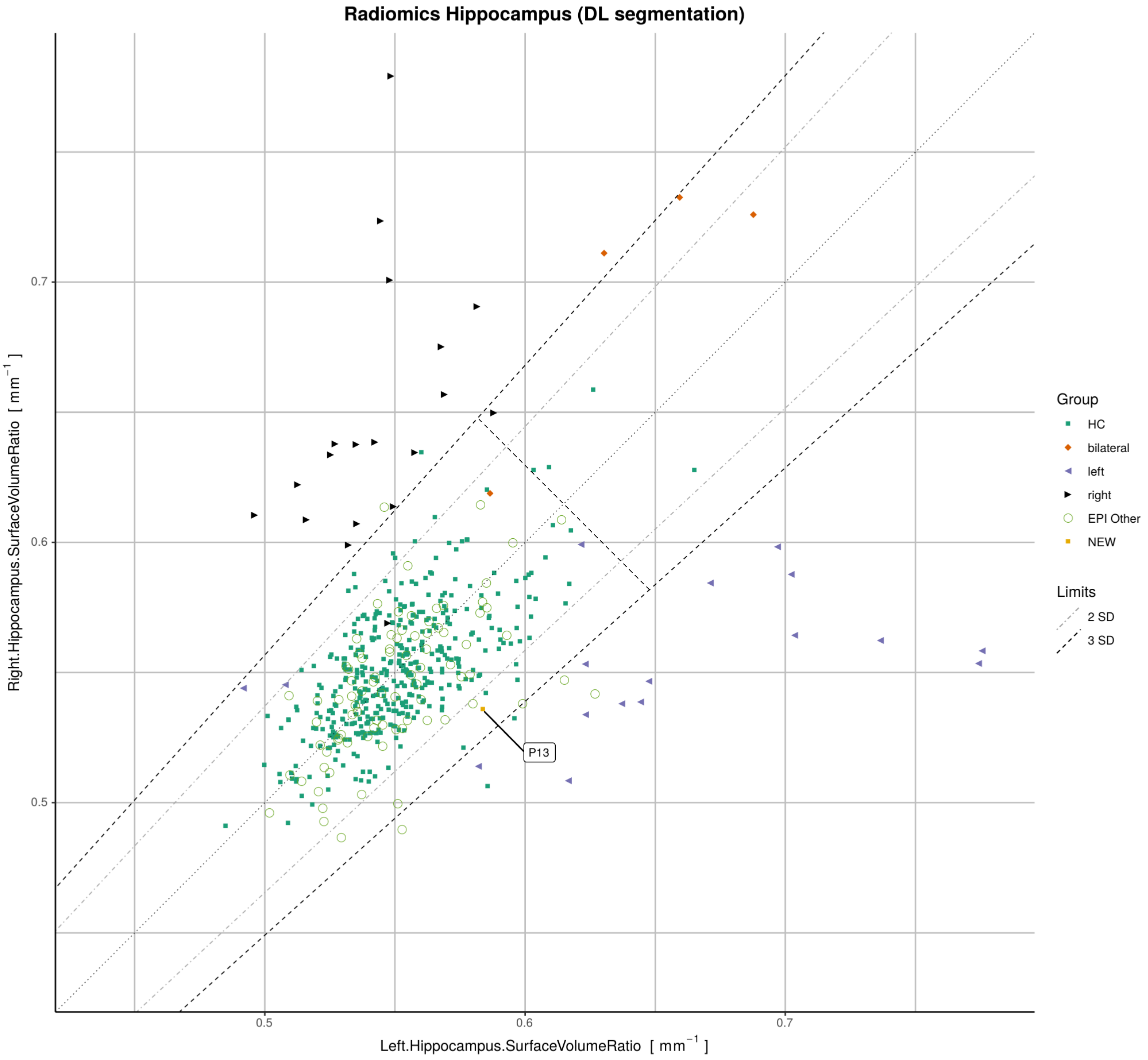

ID	Left.Hippocampus.Volume [ml]	Right.Hippocampus.Volume [ml]	Left.Hippocampus.SurfaceVolumeRatio	Right.Hippocampus.SurfaceVolumeRatio
P13	4.134	4.129	0.584	0.535

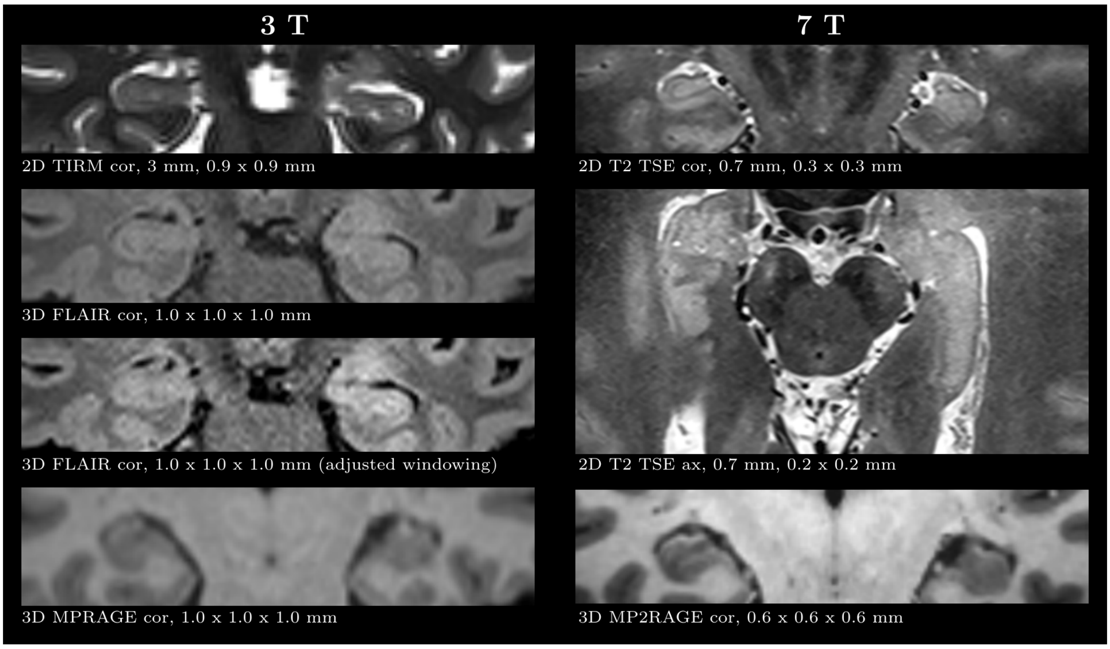

Figure S4: Example of patient *P13* with *HS left*. In the first round, 6/6 raters classified this case as *HS left*, whereas in the second round only 4/6 while the other two changed to *normal*, possibly because the QReport was between two and three standard deviations (SD). Case summary: A 50-year-old male patient with pharmacoresistant epilepsy with hippocampal sclerosis on the left and seizure onset in the left mesiotemporal region confirmed within phase I and phase II evaluation. The patient was referred to 7T MRI due to ambiguous findings on previous 3T MRI examinations and initially suspected autoimmune encephalitis, which could not be confirmed.

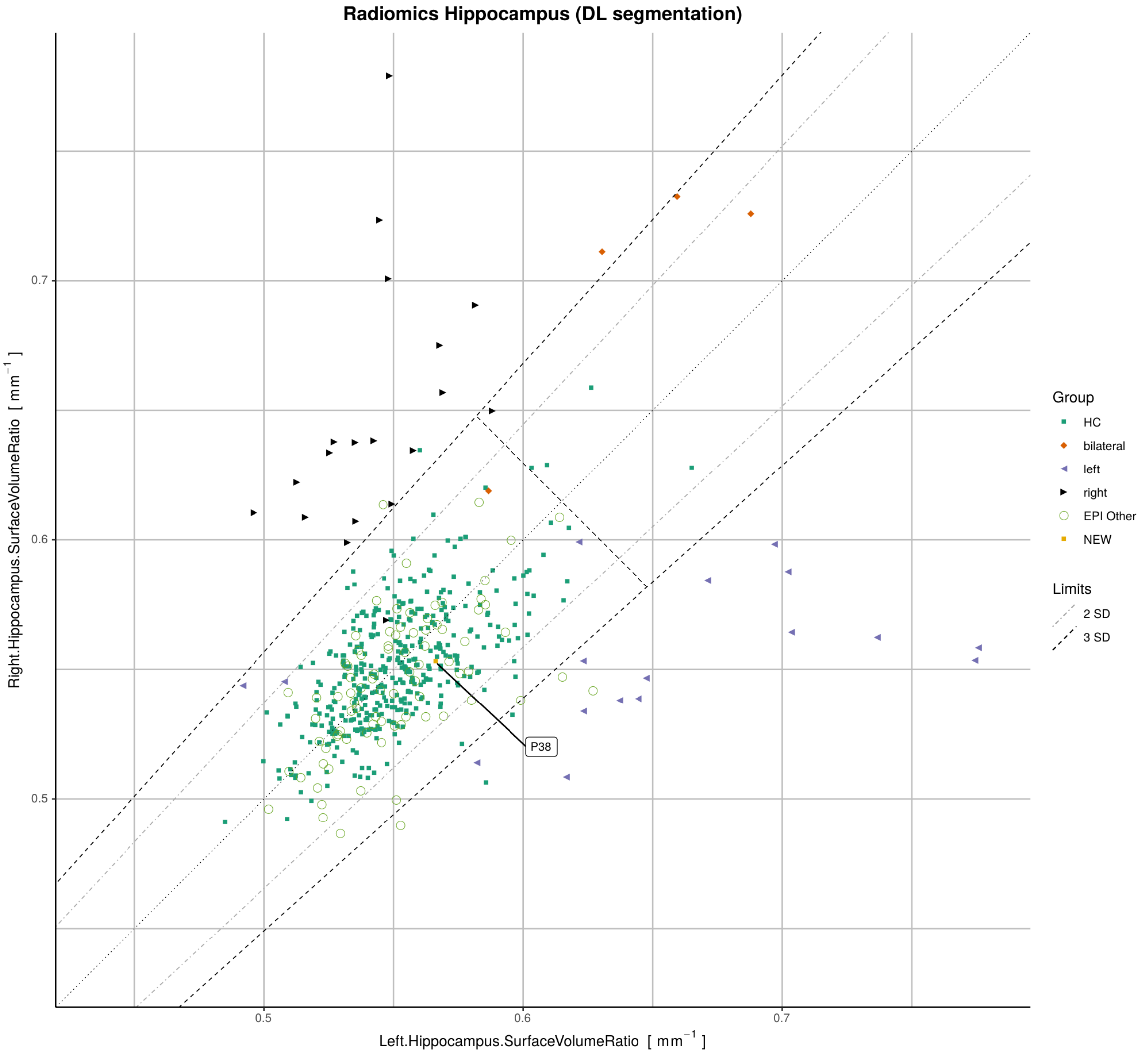

ID	Left.Hippocampus.Volume [ml]	Right.Hippocampus.Volume [ml]	Left.Hippocampus.SurfaceVolumeRatio	Right.Hippocampus.SurfaceVolumeRatio
P38	4.211	4.443	0.566	0.553

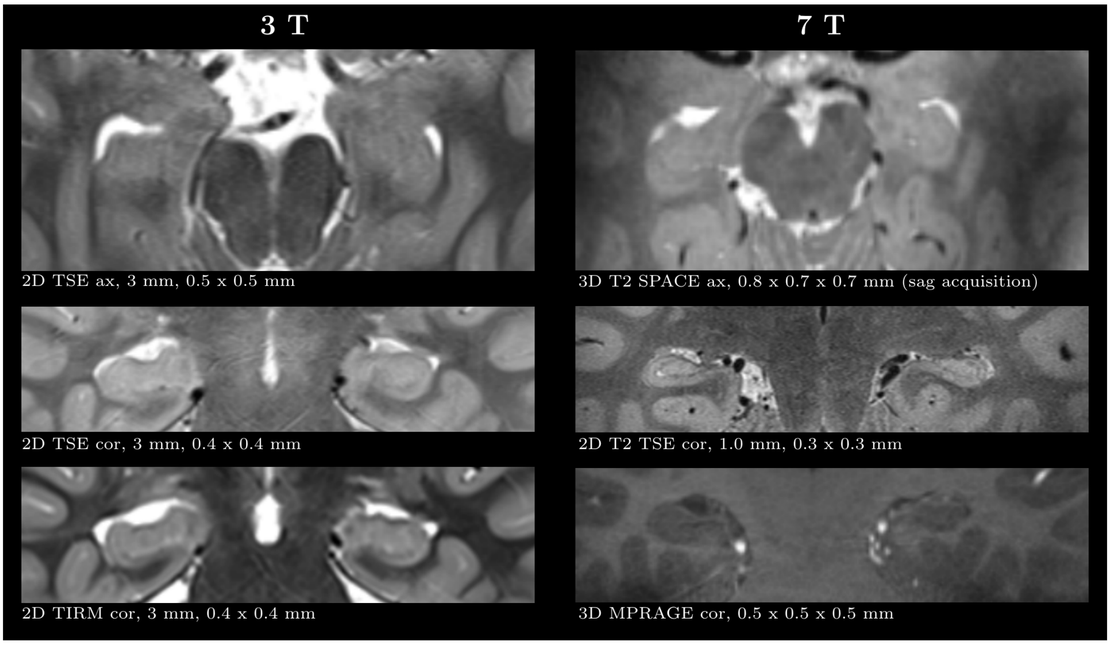

Figure S5: Example of patient *P38 without HS*. In the first round, 3/6 raters classified this case as *HS right*, whereas in the second round all rated it as *normal*.

Case summary: A 13-year-old male patient with pharmacoresistant focal epilepsy. On FDG-PET a hypometabolic area in the right temporal region was suspected. The patient was referred to 7T MRI for further clarification. In the pre-surgical phase II evaluation including electrode placement in the right hippocampus and amygdala no seizure onset zone was localized.

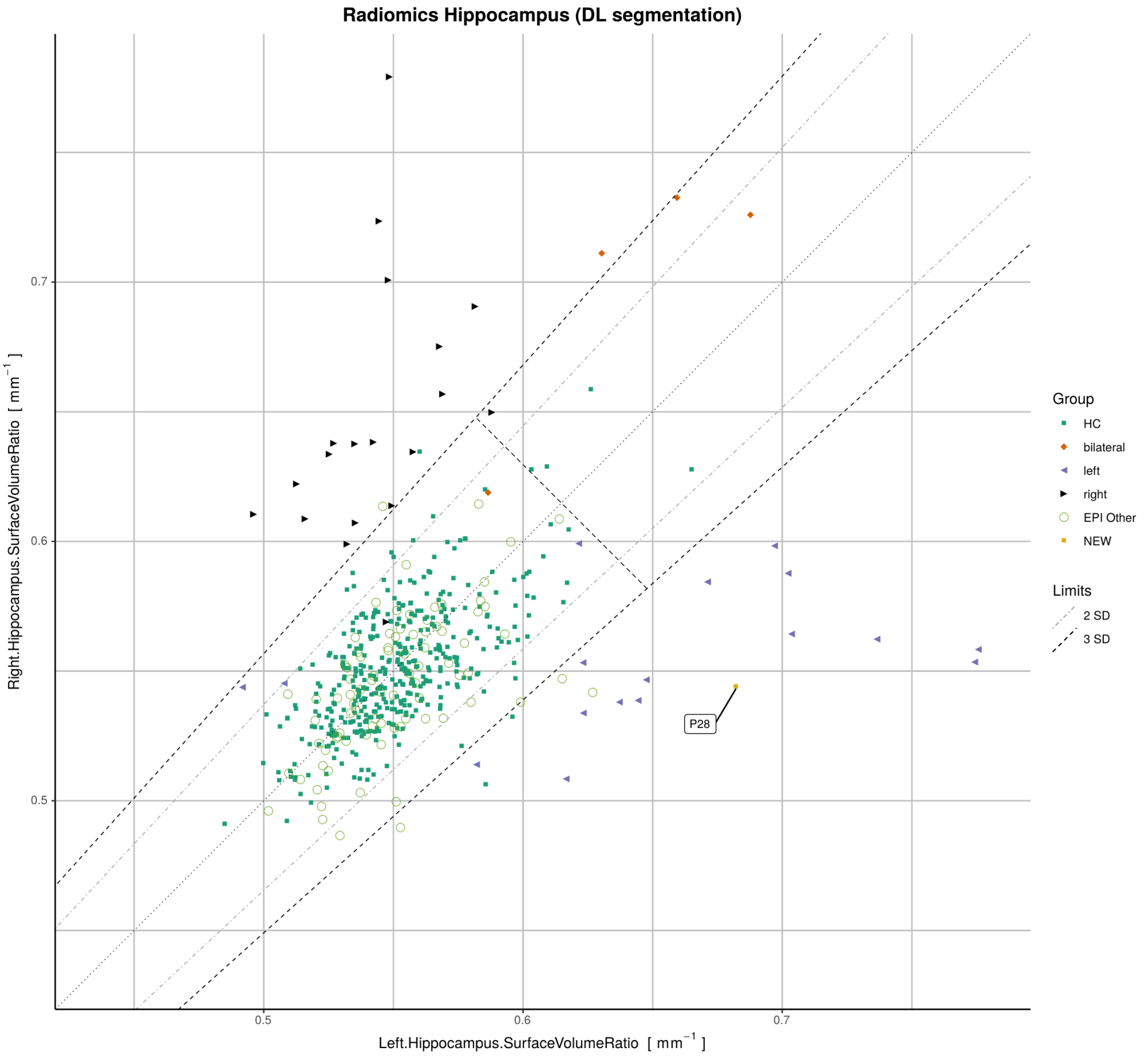

ID	Left.Hippocampus.Volume [ml]	Right.Hippocampus.Volume [ml]	Left.Hippocampus.SurfaceVolumeRatio	Right.Hippocampus.SurfaceVolumeRatio
P28	2.398	4.465	0.682	0.544

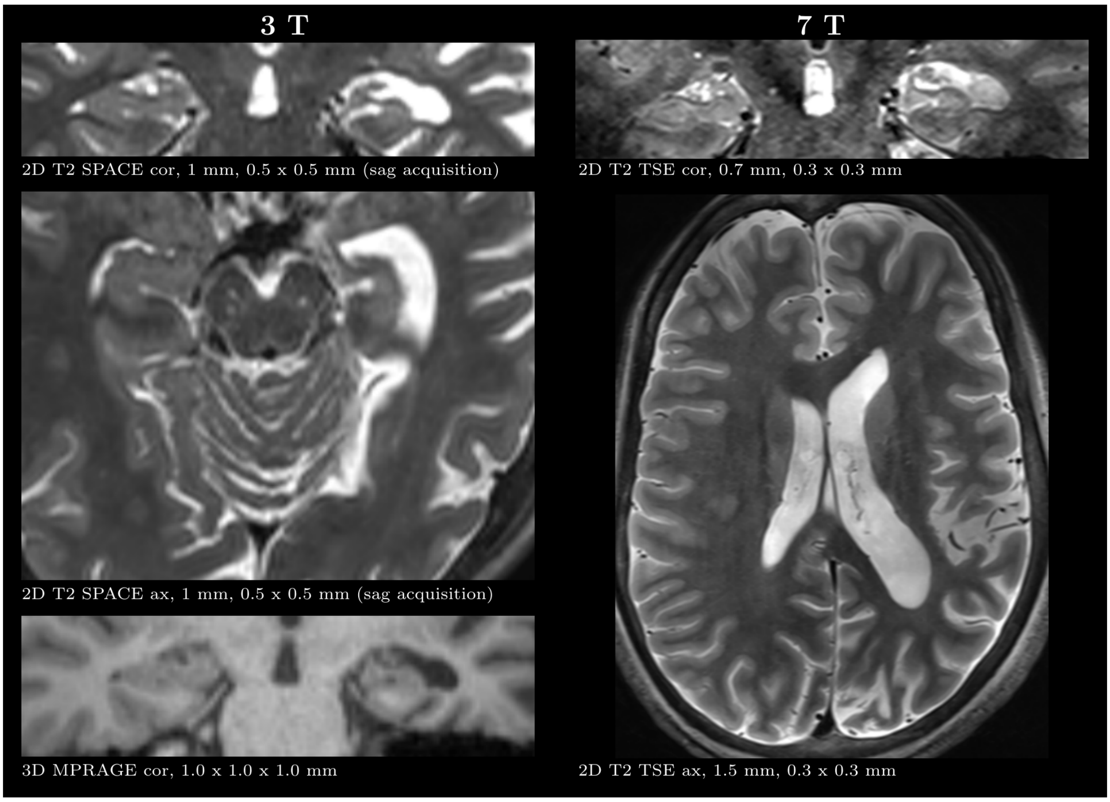

Figure S6: Example of patient *P28 without HS*. In the first round, 6/6 raters classified this as *HS left*, whereas in the second round one rater changed to *normal*. Shortened MRI protocol due to non-compliance of the patient.

Case summary: A 13-year-old male patient with pharmacoresistant multifocal temporo-occipital epilepsy left. Referred for 7T MRI for pre-surgical evaluation with the intention of palliative surgery to reduce seizure frequency. The patient presented initially with B-cell Acute Lymphoblastic Leukemia without CNS involvement and received chemotherapy according to AIEOP-BFM protocol. Six months after the start of the treatment patient experienced PRES attributed to the treatment. In MRI follow-up after 6 months, severe parenchymal volume loss in the left hemisphere, including the mesio-temporal region was described.

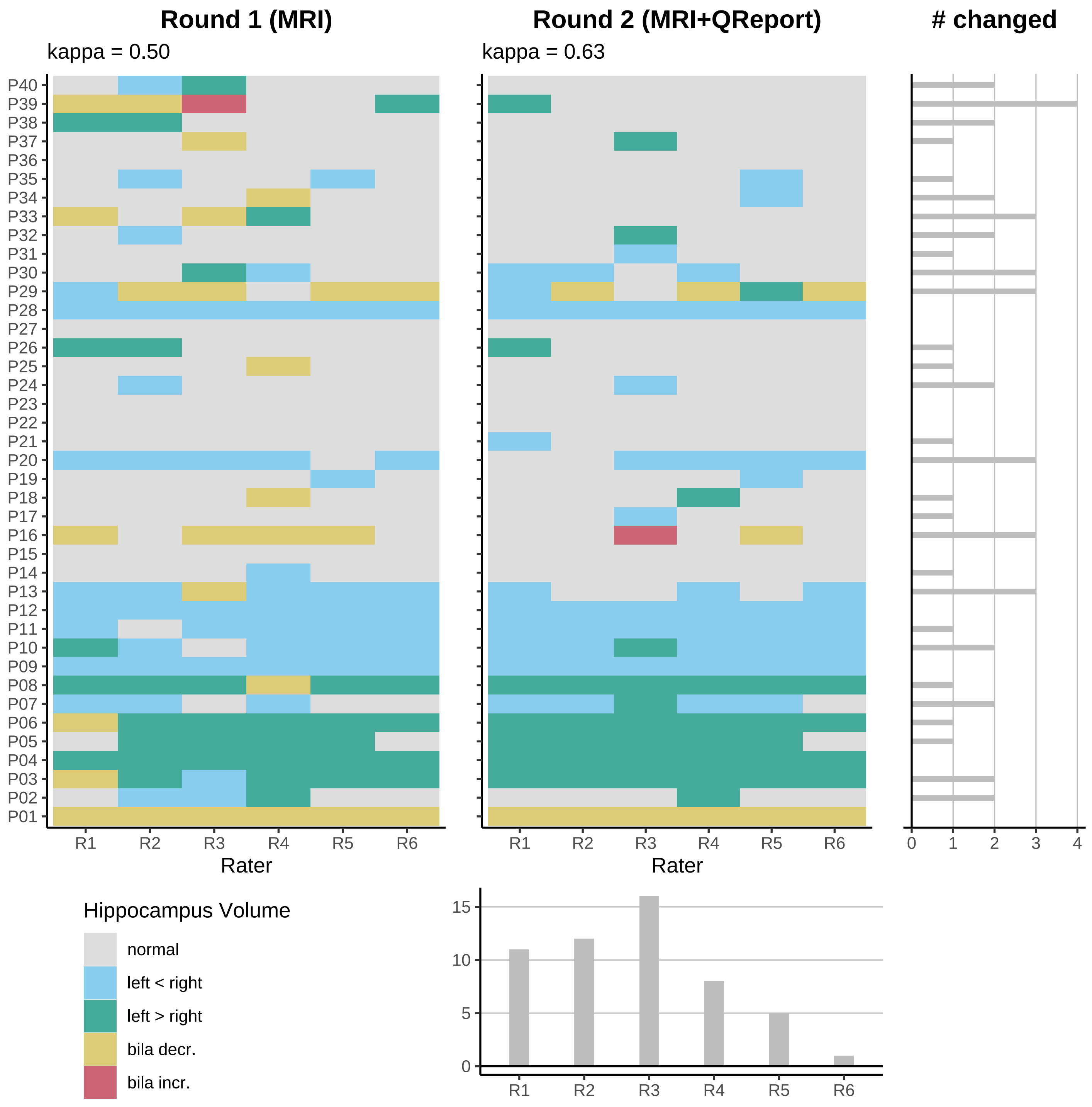

Figure S7: Visualization of all individual ratings (first two panels) for the **hippocampus volumes**. For each of the 40 cases, the number of times the raters changed their decision (# changed) is depicted. Corresponding changes per rater are shown below round 2. Neurologists (R1-R3) changed their decision more frequently than radiologists. Note: The cases were sorted by diagnosis to improve the clarity of this visualization but were presented to the raters in random order.

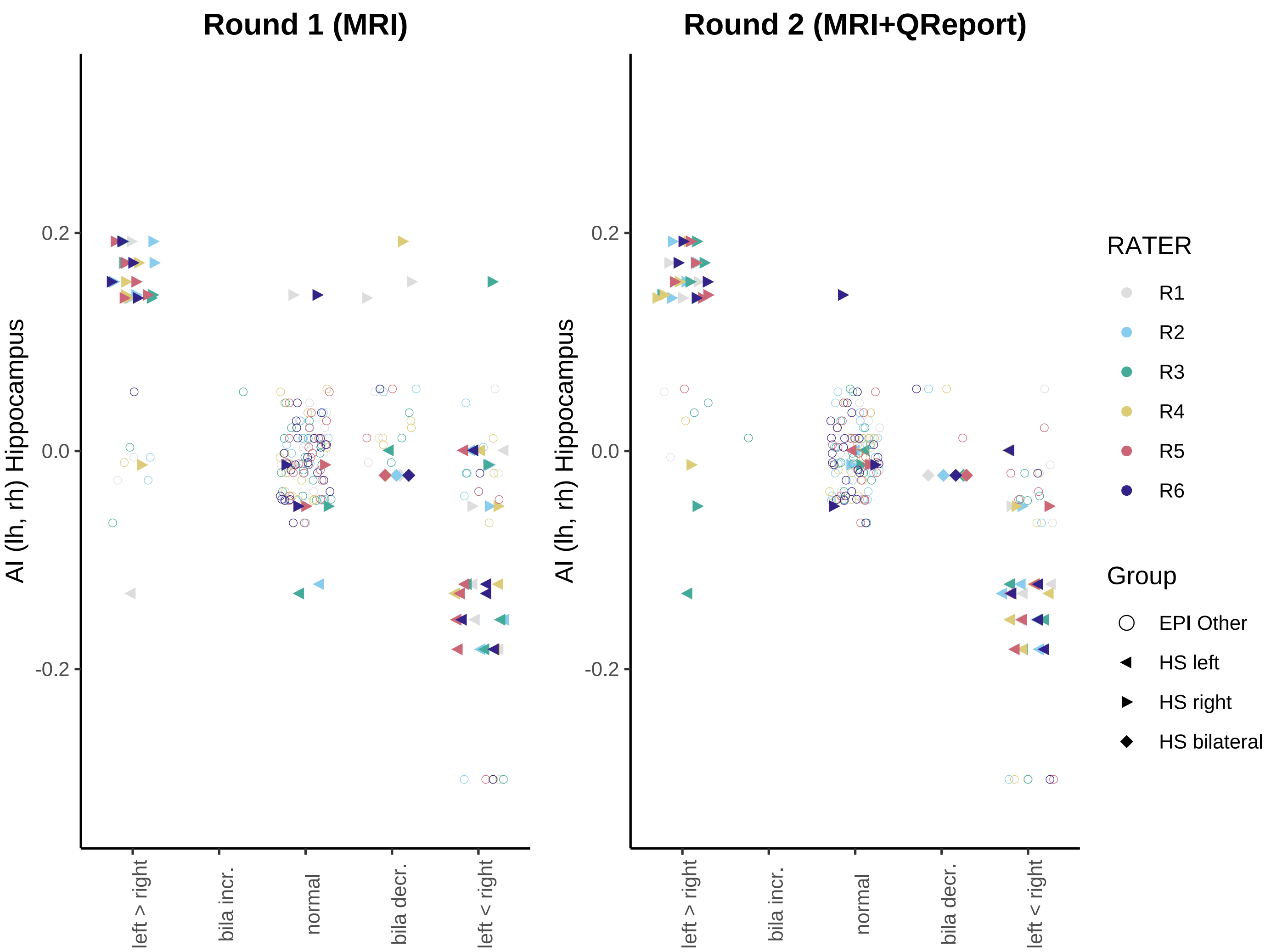

Figure S8: Ratings of the **hippocampus volumes** on the x-axis plotted against the asymmetry index (AI) on the y-axis. The symbols are shown with horizontal jitter for better readability.

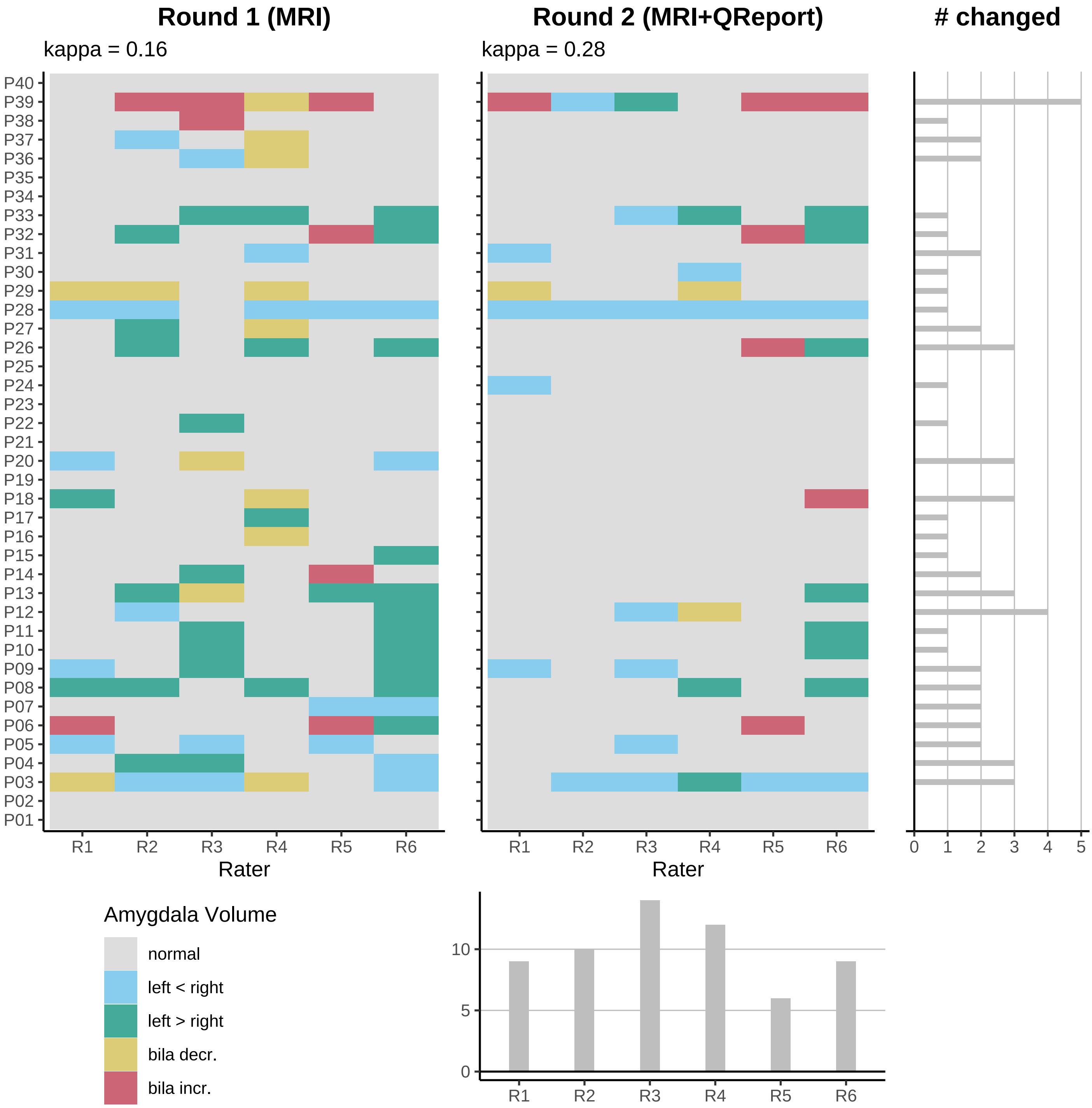

Figure S9: Visualization of all individual ratings (first two panels) for the **amygdala volumes**. For each of the 40 cases, the number of times the raters changed their decision (# changed) is depicted. Corresponding changes per rater are shown below round 2. Note: The cases were sorted by diagnosis to improve the clarity of this visualization but were presented to the raters in random order.

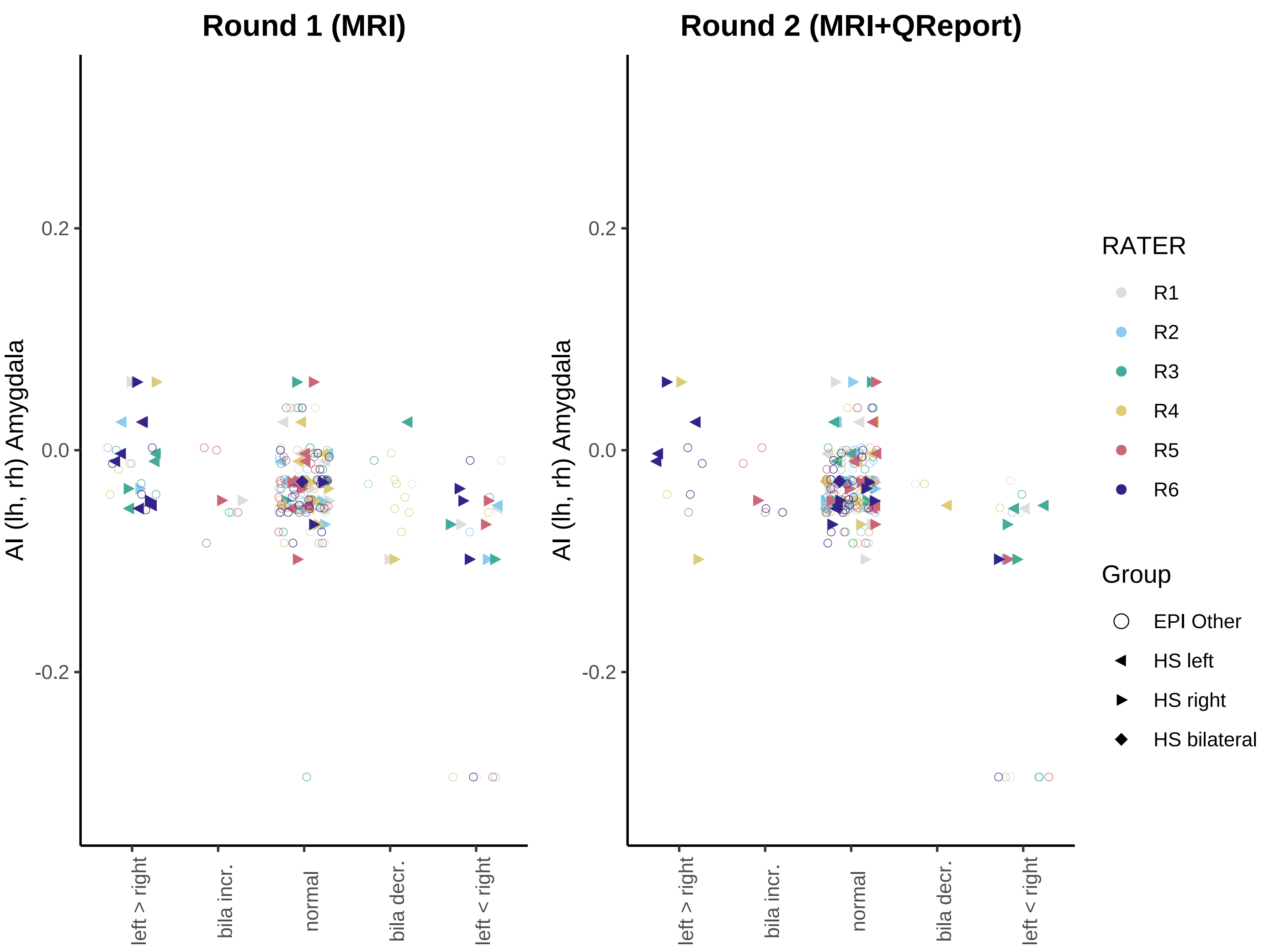

Figure S10: Ratings of the **amygdala volumes** on the x-axis plotted against the asymmetry index (AI) on the y-axis. The symbols are shown with horizontal jitter for better readability.

Round 1 (MRI)

	EPI Other	HS left	HS right	HS bilateral
EPI Other	126	1	14	1
HS left	22	29	0	0
HS right	10	0	26	0
HS bilateral	4	0	2	5

Round 2 (MRI+QReport)

	EPI Other	HS left	HS right	HS bilateral
EPI Other	149	3	14	0
HS left	12	26	0	0
HS right	0	1	26	0
HS bilateral	1	0	2	6

Figure S11: Confusion matrices of the ratings, pooled across all six raters. Their ratings appear in the rows and ground truth in the columns. In the first round, a total of 36 cases were wrongly rated as HS (22 $\times$ left, 10 $\times$ right, 4 $\times$ bilateral, whereas this reduced to 13 in the second round (12 $\times$ left, 1 $\times$ bilateral).
